# The Role of Transesophageal Echocardiography in the Intraoperative Period

**DOI:** 10.2174/157340311798220511

**Published:** 2011-08

**Authors:** Verónica Gouveia, Paulo Marcelino, Daniel A Reuter

**Affiliations:** 1 Department of Anesthesiology and Intensive Care Medicine Klinikum Itzehoe, Germany; 2 Department of Anesthesiology, Center of Anesthesiology and Intensive Care Medicine, Hamburg-Eppendorf University Medical Center, Hamburg, Germany; 3 CEDOC, Faculdade de Ciências Médicas, Lisboa

**Keywords:** Transesophageal echocardiography, hemodynamic monitoring, intraoperative, non-cardiac surgery.

## Abstract

The goal of hemodynamic monitoring and management during major surgery is to guarantee adequate organ perfusion, a major prerequisite for adequate tissue oxygenation and thus, end-organ function. Further, hemodynamic monitoring should serve to prevent, detect, and to effectively guide treatment of potentially life-threatening hemodynamic events, such as severe hypovolemia due to hemorrhage, or cardiac failure. The ideal monitoring device does not exist, but some conditions must be met: it should be easy and operator-independently to use; it should provide adequate, reproducible information in real time. In this review we discuss in particular the role of intraoperative use of transesophageal echocardiography (TOE). Although TOE has gained special relevance in cardiac surgery, its role in major non cardiac surgery is still to be determined. We particularly focus on its ability to provide measurements of cardiac output (CO), and its role to guide fluid therapy. Within the last decade, concepts oriented on optimizing stroke volume and cardiac output mainly by fluid administration and guided by continuous monitoring of cardiac output or so called functional parameters of cardiac preload gained particular attention. Although they are potentially linked to an increased amount of fluid infusion, recent data give evidence that such pre-emptive concepts of hemodynamic optimization result in a decrease in morbidity and mortality. As TOE allows a real time direct visualization of cardiac structures, other potentially important advantages of its use also outside the cardiac surgery operation room can be postulated, namely the ability to evaluate the anatomical and functional integrity of the left and the right heart chambers. Finally, a practical approach to TOE monitoring is presented, based on a local experience.

## INTRODUCTION

Perioperative hemodynamic management during major surgery can be challenging for the anaesthesiologist, especially if hemodynamic instability is likely to occur. For this reason patients are monitored in order to trace several physiological parameters. In the hemodynamically unstable patient, fluid infusion and catecholamine support are the choices of treatment, based on the information retrieved by adequate monitoring. Basic monitoring during anesthesia for surgical procedures consists of electrocardiography, pulse oximetry, non-invasive measurement of blood pressure, and, when general anesthesia is applied, capnometry and -graphy. Perhaps the most commonly used devices for advanced hemodynamic monitoring during major surgery are intravasal catheters for the continuous monitoring of arterial and central venous pressure (CVP) and, in selected cases and according to local experience, the continuous monitoring of cardiac output (CO) using transcardiopulmonary indicator dilution or arterial pulse contour analysis (PiCCO, LiDCO ,Vigileo, PRAM, and others). Pulmonary artery catheters were frequently used in the past, however, its use extremely decreased over the last decade, since several trials in the field of intensive care medicine and perioperative care could not prove any benefit for its use [1,2].

Among the new techniques for intraoperative management Transesophageal Echocardiography (TOE) is considered promising [3]. The unique ability of TOE in direct, real-time visualization of cardiac structures provides an invaluable role in the intraoperative management of cardiac surgery [4-6]. Anaesthesiologists can acquire competence in this field through specific training programmes [7].

Its systematic use in non cardiac surgery seems helpful, but publications in the field are yet scarce [8]. However, in particular in complex non-cardiac procedures, such as liver surgery or even liver transplantation its use can add important information on hemodynamic management [9]. During these procedures, hemodynamic instability frequently occurs because of surgical manipulation, and in particular because of blood loss. Wax *et al.* [10] analyzed the experience of using TOE during liver transplantation in the United States, and demonstrated that TOE was performed by 86% of anaesthesiologists in some or all liver transplant cases, although most performed only a limited scope examination. Further, only 12% of the care giving anesthesiologists were board certified regarding TOE, and only 1 centre was reported to have a policy related to credentialing requirements for TOE.

However, regarding the use of TOE in the perioperative phase, two basic questions need to be answered: First, which information needs to be gathered by an advanced monitoring device in order to enable successful management of intraoperative hemodynamic incidents; second, it needs to be analyzed, which information can be retrieved from a specific monitoring tool.

## TRANSESOPHAGEAL ECHOCARDIOGRAPHY IN CARDIAC SURGERY

In cardiac surgery, transesophageal echocardiography is used with increasing frequency for diagnostic purposes, as well as for perioperative hemodynamic monitoring. The use of TOE in adult as well as in pediatric cardiac surgery has provided a large amount of new information not previously available, and an increasing number of complex procedures also in interventional cardiology are today simply not possible without TOE.

When analyzing the literature the most frequently measured hemodynamic parameters assessed by TOE are cardiac output and left ventricular filling, i.e. preload. Further, TOE is used for functional assessment of the LV by identifying global or regional LV wall motion abnormalities, as well as for the assessment of valvular dysfunctions [11,12,13]. In valve surgery, TOE plays a particular role, because it allows assessing the immediate results of surgical reconstruction or replacement. For example, in during aortic valve replacement, TOE allows sizing the valve annulus and confirming satisfactory function after implantation.

Compared to other monitoring techniques, the use of TOE can be categorized as being very safe because of its minimal invasiveness. Kallmeyer *et al*. [14] reported the incidence of intraoperative TOE- associated complications in a retrospective case series of 7200 patients in cardiac surgery. The most common TOE- associated complication was severe odynophagia, which occurred in 0.1% of the study population. Other complications included dental injury (0.03%), endotracheal tube malpositioning (0.03%), upper gastrointestinal hemorrhage (0.03%) and esophageal perforation (0.01%) [15,16]. Kremer *et al*. studied 400 patients using TOE for up to 12 hours during elective cardiac surgery and demonstrated that TOE was associated with fewer complications than intravascular pressure and flow measurements by the use of the pulmonary artery catheter, [17].

A current literature review shows from small to large series of patients in cardiac surgery, that the use of TOE is a valuable instrument in the intraoperative period. Compared to other monitoring techniques TOE had more influence in intra-operative decision making. Bergquist *et al*. showed that TOE was the single most important guiding factor in 17% of the 587 interventions, which were assessed. Interventions involving fluid administration contributed to 47% of the total clinical decisions. TOE was the most relevant decision tool leading to fluid administration in 30% of cases, compared to only 7% by the pulmonary artery catheter in these cases. In a prospective cohort study, Mishra *et al*. [18] evaluated 5016 adult patients, 1356 underwent valve procedures and 3660 coronary artery bypass grafting. They observed that in 36% there were TOE- guided hemodynamic interventions, and in 23% TOE was the sole guiding factor initiating therapy. Pierre *et al*. [19] showed that during cardiac surgery TOE changed in 53% of the cases actual medical therapy and 30% it influenced surgical decisions. In a large series of 12 566 patients undergoing cardiac surgery, studied by Eltzschig *et al*. [20], the impact of intraoperative TOE on surgical decisions was assessed. In more than 9% intraoperative procedures TOE influenced cardiac surgical decisions.

According to the literature TEE is obviously advisable in cardiac surgery. But available literature also confirms the need for specific training for its use in the perioperative phase.. Guidelines are well defined by the American Society of Echocardiography (ASE) and the Society of Cardiovascular Anaesthesiologists (SCA) [7].

## TRANSESOPHAGEAL ECHOCARDIOGRAPHY FOR NON CARDIAC SURGERY

The questions faced by anaesthesiologists during non cardiac surgery are rather different. Hemodynamic instability and rapid changes in volume status (mainly due to acute blood loss) are the most serious complications and challenges for perioperative management.

Most frequently, hemodynamic instability is associated with hypotension and/or arrhythmias. Episodes of hypotension are dangerous. Organ perfusion is rapidly impaired, and intra and post operative complications may appear as result of organ ischemia. Some organs are more sensitive to ischemia, as the case for the central nervous system or the kidneys.

The main issue is the rapid recognition of these life threatening situations, and identification of their causes, which is the basis for adequate treatment [21]. The pathophysiology of hypotension, regardless of aetiology, may be due to cardiac (pump) failure, hypovolemia, or reduced peripheral vascular resistance. For discrimination, one usually needs to determine cardiac output, vascular filling and peripheral vascular resistance. As peripheral vascular resistance is dependent on cardiac output, a normal or increased cardiac output in a patient with hypotension usually indicates a reduced peripheral resistance. So, cardiac output in this setting can be discriminative, identifying patients needing vasopressor therapy. However, unfortunately, at the bedside, pathophysiology is frequently not so easy to recognize because different hemodynamic aberrations can be present in parallel. Even in acute blood loss, reduced peripheral resistance can be present, as for example due to systemic inflammation, along with signs of hypovolemia. Therefore, the assessment of cardiac output and cardiac preload play a central role in advanced intraoperative monitoring.

Transesophageal echocardiography can be helpful and add important information. In experienced hands, the correlation between echocardiography and thermodilution for measurement of cardiac output is generally acceptable [22-23]. Although the focus so far was on the evaluation of cardiac output monitoring by echocardiography, the impact of direct visualization of cardiac structures has so far not extensively studied in non-cardiac surgery. But of course, also in the perioperative care for non-cardiac surgery, for example monitoring left ventricular function, allowing the detection of pump failure, or visualization of right heart chambers, allowing the real-time diagnosis of pulmonary hypertension and overload (pulmonary thromboembolism) can be potentially life-saving. Other advantages can become evident with the regular use of the technique.

In the following, the basic cornerstones of hemodynamic assessment by TEE are discussed.

## ASSESSMENT OF CARDIAC OUTPUT

Monitoring of CO has gained particular attention as it is one main determinant of oxygen transport. But if circulatory flow is adequate to fulfil the needs of the end organs, can of course not be determined by its measurement. Perhaps the main use of monitoring CO is to detect changes in this variable over time, especially during episodes of hemodynamic instability and following therapeutic interventions. Considering this main function of CO monitoring, the changes are more important than the absolute values. Other variables provided by the continuous CO monitoring systems, such as the stroke volume variation and systemic vascular resistance index can be also provided directly by TOE. However, systemic vascular resistance can be easily derived from the CO and CVP.

Nonetheless CO alone can not be considered a surrogate for left ventricular function. It depends not only on the left ventricular stroke volume and heart rate, but it is also strongly load-dependent, i.e. cardiac filling (preload) and peripheral vascular resistance (afterload). This means that the sole information on CO does not give an accurate picture of a specific situation. The only exception may be the presence of a normal or high CO in patients with a mean blood pressure lower than 60 mmHg, indicating a low peripheral vascular resistance. However, left ventricular performance is still undetermined.

The gold standard for CO determination are direct measurements of blood flow in the ascending aorta by electromagnetic or ultrasonic flow probes mounted around the aorta (experimental gold standard), or, in the clinical setting, using the Fick principle. However, this technique requires a controlled airway to assess in- and expired gas concentrations. Further, patients need to be hemodynamically stable during the measurement period.

The most common technique used at the bedside is the thermodilution method, an application of the indicator dilution principle, in which the indicator is cold saline [25]. Actually, for many authors this method represents the clinical “gold-standard”, due to its extensive use in the past, often leading to ignorance to methodological limitations of this technique. Further, serious concerns have arise on the routine use of pulmonary artery catheterization in several settings, especially in the critically ill. The available devices for continuous CO measurements, based on dilution methods, have been introduced in medical practice in the recent years [26,27].

## MEASUREMENT OF CARDIAC OUTPUT BY ECHOCARDIOGRAPHY

Echocardiography has been studied for several years as a non invasive tool for CO determination. Several technical approaches have been used, both using the transthoracic and transesophageal approach. The comparison between echocardiographic methods for CO evaluation and the more frequently used other monitoring techniques, such as thermodilution is discussed here. Two main approaches of echocardiography are used. The first is provided by esophageal probes, placed on the descending aorta. Pulsed-wave analysis is used, which, after correction for the CO loss in the upper branches of the aorta (usually standardized to 30%), provides a continuous CO determination. These probes are “blind”, i.e. they cannot provide a visualization of cardiac structures. Other methods rely on the classic TOE views, assessing the CO either through Doppler analysis in several places (mitral orifice, left ventricular outflow tract). Considering methodology, they can be divided in Doppler derived measurements, and volumetric assessment of heart chambers. The volumetric approach is based on the determination of end-systolic and end-diastolic ventricular volumes, through a short axis transgastric view, calculating the stroke volume.

Bettex *et al*. [28] studied 30 patients undergoing cardiac surgery. Several echocardiographical Doppler derived methods were used, namely transmitral, transpulmonary, right and left outflow tract, as were area calculations using transgastric views (Simpson´s rules). The comparator was pulmonary artery thermodilution. Overall a low agreement was observed between the methods. The best echocardiographical method was the Doppler based flow assessment over the left ventricular outflow tract, assuming a triangular shape of valve opening. Stoddard *et al*. [29] evaluated patients under mechanical ventilation in the ICU, using the transgastric view in order to obtain the pulsed-wave Doppler of the left ventricular outflow tract. A good correlation was found between echocardiography and a simultaneous measurement of CO using thermodilution. Estagnaise *et al*. [30] performed a study in 22 mechanically ventilated patients, and the echocardiographical method used was the CO obtained in a single plane of the mitral valve. A total of 74 measurements were performed, and a significant correlation was found (r=0.78). More importantly, changes in CO were similar by both methods, concluding that TEE derived CO was capable to trace its changes. Su *et al*. [31] compared bolus thermodilution and continuous thermodilution methods for CO measurement with Doppler derived esophageal CO, in 24 patients undergoing coronary artery bypass graft surgery. A better agreement was found between continuous thermodilution and esophageal CO (0.84) and was poorer between bolus thermodilution and esophageal CO (0.406). Axler *et al*. [32] compared a Doppler derived method and a volumetric method of CO determination, comparing both to thermodilution. The best method was the Doppler derived one, especially in the group of patients with sepsis (n=40 out of 55 patients enrolled). The authors concluded that the best option is the left ventricular outflow tract pulsed Doppler CO. Dark *et al.* [33] performed a meta-analysis on the comparison of 2 available ultrasonographic devices, which use the analysis of the pulsed-wave Doppler of the descending aorta. These devices are the CardioQ and HemoSonic 100. Left ventricular stroke volume is estimated by mean systolic velocity measurement using a nomogram in the CardioQ, and an M-mode echocardiography estimate of aortic diameter using HemoSonic 100. Twenty-one studies analysed CardioQ, and 3 Hemosonic 100, both comparing to thermodilution using a pulmonary artery catheter. In total, these studies involved 314 patients and 2400 paired measurements. A good correlation was found between both methods, with a mean difference of -0.69 to 2.00 l/min. The authors concluded that there is a good agreement between both echocardiographical methods and thermodilution CO. Gola *et al*. [34] analysed the accuracy of Doppler measurements in low output states, as in patients with congestive heart failure. A total of 73 patients who underwent evaluation for cardiac transplantation with dilated cardiomyopathy were evaluated. Simultaneous right heart catheterization with determination of CO through the Fick method, and a transthoracic echocardiogram with CO determination using the left ventricular outflow tract pulsed-Doppler analysis were performed. The correlation between thermodilution and Fick was 0.81, and 0.90 for the Fick method vs. echocardiography. In patients with significant tricuspid regurgitation (n=34) the r value for the agreement between the Fick method and thermodilution was 0.68, and 0.86 for the agreement between echocardiography and the Fick method. The authors concluded that the presence of significant tricuspid regurgitation influences significantly the accuracy of thermodilution CO. Also acceptable agreements between CO measurements with transcardiopulmonary thermodilution and echocardiography were described [35]. In a recent study performed by our group in liver transplant patients [36], we observed an agreement between CO determined by transthoracic echocardiography using the pulsed-wave Doppler analysis of the left ventricular outflow tract and simultaneous bolus thermodilution of 97% (r=0.97). The higher differences were observed in high output states (patients with cirrhosis), which is in accordance to earlier reports, linked to the limitations of pulsed-wave Doppler to detect higher flow velocities. Also, a significant number of patients were hypothermic immediately after surgery, with central temperatures frequently between 32 to 34 degrees Celsius, which increases errors in measurement of the thermodilution techniques. Similar results in liver transplant patients were described by Boucaud *et al*. [37]. Comparing the new techniques for CO assessment, Nissen and co-workers [38] compared CO obtained through arterial pulse contour analysis and thermodilution in liver transplant patients. They also described a good agreement between the techniques.

Another method to determine CO using TOE is the measurement through the mitral pulsed-Doppler [40]. In this method mitral valve annulus is used as a surrogate for cross-sectional area. The accuracy of determining stroke volume through the mitral valve has not been accurately investigated so far. The mitral valve orifice does not have a perfect geometrical shape, and it is therefore not the preferred structure for CO determination. Data published on this technique are solely from the transthoracic approach. However, one limitation of all echocardiographic techniques for CO determination is the operator dependency, which includes both intra-observer variability, as well as inter-observer variability. This further stresses the need profound and continuous education and training, when using these techniques.

## ASSESSMENT OF VOLUME STATUS AND GUIDING VOLUME THERAPY

The assessment of volume status is a cornerstone of perioperative hemodynamic management. Monitoring filling pressures, i.e. central venous pressure and pulmonary artery occlusion pressure (PAOP) were the “standard” methods used for decades. However, these static, pressure-based parameters are now under serious criticism. Past concepts of fluid and volume therapy in several contexts, including the intraoperative period, based on the achievement of higher than normal physiologic filling pressures. By that, adequate volume status, and therefore maintenance of adequate cardiac output should be assured [42]. But the limitations of these parameters are now well characterized. Cardiac filling pressures do not allow predicting the increase in intravascular volume and cardiac output [43,44,45]. This means that a low filling pressure can not indicate that volume administration will lead to an increase in intravascular volume or cardiac output. Several factors contribute to this inability of the filling pressures to serve as reliable parameters of preload or volume responsiveness, which includes their dependency on cardiac compliance (i.e. diastolic properties of the heart), on changes in intrathoracic pressure during mechanical ventilation, on the hemodynamic consequences of positive end-expiratory pressure (PEEP) or intra-abdominal pressure. Interestingly, the concept of using filling pressures as parameters of cardiac preload and volume responsiveness was even questioned in healthy individuals [46]. The actual concepts of volume responsiveness use a set of parameters that can accurately predict an increase in CO, due to volume administration. These are the so called dynamic parameters of volume responsiveness [43,47].

However, also the global question, whether fluid restriction or a more liberal approach of perioperative fluid and volume therapy should be aimed, has extensively been discussed within the last decade. In sepsis, a situation characterized by an increased leak of fluid to extravascular space, early and “goal-directed” hemodynamic stabilization, i.e. also in particular volume loading in the early phase is associated with improved outcome. In the recent years several groups of investigators had provided evidence that in the perioperative period, an excess of fluid can be harmful in particular because of edema formation [48]. More recently, Brandstrup *et al*. [49] conducted a study of 172 patients, monitoring the weight gain in the postoperative period of colorectal surgery. In the patients subjected to restricted fluid administration, assessed by weight gain, a lower number of complications could be observed, including cardiopulmonary (7% vs 24%), and tissue-healing (16% vs. 31%) complications. The same findings were observed by Walsh *et al*. [50], in which the authors describe a decreased number of complications after major gastrointestinal surgery, mostly performed in ASA 2-3 patients. Further, Adesanya *et al*. [51], described a beneficial effect in a cohort of 41 patients undergoing vascular surgery. In liver transplant patients the beneficial effect of a restrictive fluid regimen is also described by Jiang *et al*. [52]. In 62 patients, these authors observed that a negative fluid balance <500ml in the first 3 days after surgery was independently associated with post-operative complications. Also the effect of the amount of fluid in different organs and healing process was studied. Marjanovic *et al*. [53] demonstrated that the amount of crystalloid infused influenced the intestinal anastomotic stability. In a rat model they observed a bursting cardiac output in the high volume group as well as tissue oedema. Reports of such oedema and bowel swelling are often observed in our patients, impeding surgery, and increasing the probability of complications. In an animal model, Hiltebrand *et al*. [54] studied tissue oxygen pressure in the small bowel and colon under different fluid regimens. In this model, no difference was found, and the authors suggest efficient auto-regulation of intestinal blood flow. Ozmen *et al*. [55] also studied the effects of large fluid infusion in an animal model, observing the deleterious effects of larger amounts of extravascular fluids. The influence of excessive fluid administration in several tissues was also confirmed by other authors [55-62].

Several meta-analyses support the previous data. Rahbari *et al*. [62] observed that a restrictive and goal-directed fluid therapy rather than standard regimens are linked to a better outcome in colorectal surgery. However, the decisive point is that infusion regimens within the single studies were not comparable. What was defined in one study to be “fluid restrictive”, was already “liberal” in other studies [63], and vice versa [61], making the final drawing of conclusions out of these studies difficult [64]. Further, the type of fluid used for a) fluid replacement (i.e. compensation of extravascular and intracellular fluid deficits), and b) intravascular volume deficits (i.e. hypovolemia due to blood loss) has to be taken into consideration, when analyzing these data [65]. On the other hand, an increasing number of studies within the last decade repeatedly gave evidence that early and goal-directed hemodynamic optimization in the perioperative phase with the aim to optimize stroke volume and cardiac output mainly by optimizing cardiac preload (i.e. volume loading) reduces postoperative complications [66-73]. These findings were recently strengthened by a meta-analysis conducted by Hamilton *et al*. [74]. Those authors showed that a pre-emptive strategy focusing on interventions targeted to optimize major hemodynamic parameters (i.e. stroke volume and cardiac output) in fact can reduce not only postoperative morbidity, but also mortality. However, it needs to be pointed out that in all of these studies, the defined end-points of hemodynamic optimization (hemodynamic goals) were not uniform. Even where hemodynamic parameters like CO output or oxygen delivery were evaluated, an increase in these parameters cannot be directly be indicative of a positive influence. Further, originally the dynamic parameters of volume responsiveness were described to optimize preload in the hemodynamically unstable patient, i.e. in patients with low cardiac output and/or hypotension. Therefore, additional volume loading in a hemodynamically stable patient free of catecholamine support just because of the presence of a parameter indicating volume responsiveness may potentially lead to non-necessary volume- fluid application with the risk of edema formation.

## TOE AND DYNAMIC CONCEPTS OF FLUID INFUSION IN THE OPERATING ROOM

By far the most assessed echocardiographical technique for assessment of volume status is the evaluation of the inferior vena cava, i.e. its diameter as a “static” parameter of preload, as well as its ventilation-induced changes, reflecting a “dynamic” parameter of volume responsiveness. In several settings, this was found to be easy to assess, and effective in guiding fluid infusion [75-78]. But its main limitation for perioperative use is that it is although recently described by the use of TEE, mainly a transthoracic echocardiographical approach [79]. As transesophageal echocardiography is the main tool in the operating room, parameters derived from this approach are more extensively studied. Here, the most commonly used parameter for left ventricular preload assessment is left ventricular end-diastolic area (LVEDA). However, LVEDA is also a static assessment of preload, not allowing assessing volume responsiveness [43]. However, assessment of volume responsiveness by functional parameters based on heart-lung interactions under mechanical ventilation is also possible with echocardiography, mainly by techniques using pulsed-wave Doppler within the left ventricular outflow tract or the descending aorta. Using TOE, the left ventricular outflow tract is assessed by deep the transgastric view. The validity of aortic flow measurements and its ventilation induced variations by transesophageal Doppler was broadly evaluated vs. the more widely used systems of continuous cardiac output measurement within the last years, however with varying results. Biais *et al*. [80] found acceptable limits of agreement between stroke volume variation derived by arterial pulse contour analysis using the FlowTrac/Vigileo device and transesophageal aortic flow Doppler. In contrast, Belloni *et al*. [81] showed that there was not a good correlation between pulse contour derived stroke volume variation using the LiDCO device and transesophageal parameters, including aortic flow-derived stroke volume variation and end-diastolic and end-systolic left ventricular areas. This discrepancy could also be impressively demonstrated in a small series of cardiac surgery patients, in which both, Doppler derived measurements of beat to beat stroke volume, and those derived from a peripheral (radial artery) arterial pulse contour signal were compared against measurements with a flow probe placed around the aorta (i.e. gold standard measurements) under acute changes in preload [82]. Also Gouvea *et al*. [83], reported in 15 patients undergoing liver transplantation that pulse pressure variation, proclaimed as one parameter for preload optimization in Doppler-guided hemodynamic strategies, failed to assess volume responsiveness in this group of patients.

Besides this ongoing discussion upon accuracy of the less invasive approaches of cardiac output monitoring by pulse contour analysis and Doppler techniques, a multitude of clinical outcome studies using these beat-to-beat monitoring devices have been published within the last years. Diaper *et al*. [84] used esophageal Doppler monitoring to guide fluid therapy in 127 high-risk patients undergoing lung surgery due to cancer, concluding that this approach may valuable for this purpose. Goddard *et al.* [85] studied 128 patients undergoing colorectal surgery and compared CVP-guided and Doppler-guided fluid infusion. They found that in the Doppler-guided fluid therapy arm there was a lower hospital stay and, by authors’ judgement, a more preferable hemodynamic situation reflected by higher oxygen delivery and cardiac output. In another setting, where fluid infusion is critical, Benes *et al*. [86] evaluated a Doppler-guided protocol in multiple trauma patients, concluding that these patients presented lower levels of blood lactate. Walsh *et al*. [87] performed a meta-analysis of 4 studies, 393 patients undergoing abdominal surgery, comparing Doppler-guided fluid administration and standard fluid infusion. In the arm of Doppler guided fluid therapy there were fewer postoperative complications, shorter hospital stay, but the amount of fluid infused did not differ significantly. As the authors pointed out a cost-effective analysis between Doppler guided therapy and restrictive fluid regimens is required. In another meta-analysis performed by Abbas *et al*. [88], the same findings are described, including a beneficial effect of Doppler-guided fluid therapy in early outcomes in patients undergoing abdominal surgery. However, CO and central hemodynamic parameters could not be the sole parameters in guiding intra operative therapy in the future [89].

## LEFT VENTRICULAR FUNCTION ASSESSMENT

There is a large amount of literature regarding the use of echocardiography for risk stratification before surgery [90,91]. But its use for the assessment of both left and right ventricular performance in the operating theatre besides for cardiac surgery procedures has not been broadly evaluated so far. However, the possible additional advantages of the perioperative use of transesophageal echocardiography, i.e. the anatomical and functional evaluation of cardiovascular structures can often have clinically highest relevance. An excellent example is the report by Cavallaro *et al*. [92], who described systolic anterior wall motion (SAM, dynamic left ventricular obstruction) causing hemodynamic instability. Although a feature of hypertrophic cardiomyopathy, in conditions of extreme hypovolemia, as can be found in severe acute haemorrhage, it may cause relevant hypotension, which can be corrected by volume infusion. Interestingly, this is phenomenon not restricted to patients with left ventricular hypertrophy.

However, several issues need to be discussed, when transesophageal echocardiography should be recommended as a “monitoring device” on a broader basis in the perioperative phase. It is obvious that complex, time consuming methods, requiring assessment of multiple single parameters or equations will not be helpful in an acute setting of hemodynamic instability in the operating theatre, where immediate therapeutic consequences are needed. Skiles [93] discussed several methods for the assessment of left ventricular performance. However they all rely on complex measurements or require specific software for analysis. Perrino *et al*. [94] described an automated echocardiographical analysis method for serial intraoperative measurements in a study conducted in 16 patients undergoing non cardiac surgery. However, clear recommendations on the structured use of transesophageal echocardiography in non-cardiac surgery are scarce.

In the following we describe a standardized approach implemented at Hospital Curry Cabal, Lisbon, for routine assessment of biventricular performance in patients undergoing major non-cardiac surgery, mostly liver surgery and liver transplantation.

## AN EXPERIMENTAL PROTOCOL

In Hospital Curry Cabral, a public institution in Lisbon, an experimental protocol was used to establish the feasibility of TOE with direct visualization of cardiac structures as a monitoring tool in patients undergoing major non-cardiac surgery. In a first step, the three clinically most relevant items of echocardiographic evaluation for perioperative care were defined. This was the assessment of CO, of volume status (and its changes as in particular seen in acute blood loss), and of left ventricular performance.

The usual monitoring tools were the continuous monitoring of arterial pressure and central venous pressure, and, in selected cases, additionally the continuous monitoring of cardiac output using PiCCO system.

We defined a standardized study protocol for TOE assessment, which had to be used perioperatively. It should be on the one hand easy enough to be applied during clinical routine, but on the other hand should also provide the care giving anaesthesiologist clinical relevant and reproducible information on cardiac function. On that basis the following sets of measurements were chosen: First the left ventricular influx by evaluation of the mitral E/A ratio in the 4-chamber view was analysed. Further, left ventricular CO was assessed by measuring the mitral velocity time integral (VTI), calculating the stroke volume index (SVI) and multiplying it by heart rate Fig. (**1**). Necessary information upon the width of the mitral valve orifice was measured in the same view Fig. (**2**). In the same 4-chamber view a measurement of the external mitral annulus systolic excursion (MAPSE) was registered Fig. (**3**). Then the probe was slightly retracted, in order to visualize the left atrium (LA), and to determine diameter and area of the left atrium in the aortic valve plane Fig. (**4**). Afterwards, the probe was repositioned for the assessment of the right atrium and ventricle. In particular the right atrial area was measured Fig. (**5**). Finally, the probe was slightly retracted, in order to visualise the superior vena cava (SVC), where the respiratory changes were measured (maximum and minimum dimensions, and their ratio; the SVC index was calculated: maximum dimension – minimum dimension x 100 / maximum dimension) Fig. (**6**). Those hemodynamic assessments using TOE had to be performed every 15 minutes or whenever considered necessary.

Data from 17 patients was gathered, submitted to liver surgery, 6 of them to liver transplantation. Here we found a good correlation between CO obtained with PiCCO system using transcardiopulmonary thermodilution and mitral valve CO (r=0.84, Figs. (**7** and **8**)); acute blood loss causing hypotension and requiring red packed cells transfusion, was earlier detected by changes in LA dimensions than by changes in CVP; further, TOE allowed to directly assess left ventricular performance The use of MAPSE as a monitoring parameter for left ventricular performance has been previously validated in several studies [95,96].

This represents first preliminary experiences with such a standardized protocol for perioperative TOE examination. Further data are needed from a larger number of patients in a prospective study to evaluate in more detail the usefulness of TOE monitoring during major non-cardiac surgery. This also comprises assessment of acute right heart dysfunction, as seen for example in acute pulmonary embolism. Also, the use of TOE is not free from potential complications, although the reported rate of occurrence is low [97,98]. In the case series described here, its use was safe with no complications. Only in one patient, the introduction of the probe needed to be facilitated by the use of a laryngoscope. Further, the assessment of calculated parameters, such as CO, could be used in an automated mode, which would further facilitate clinical utility.

In conclusion, TOE with a direct visualization of cardiac structures has the potential to provide important information on cardiovascular function during non-cardiac surgery, relevant to hemodynamic management. Its widespread use and increasing experience intraoperatively is desirable.

## Figures and Tables

**Fig. (1):**
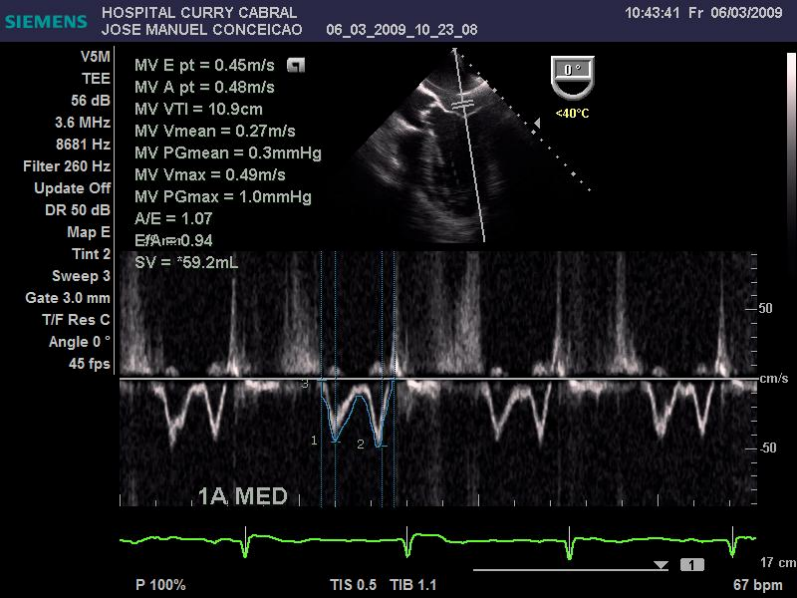
Measurement of the left ventricular influx and cardiac output.

**Fig. (2):**
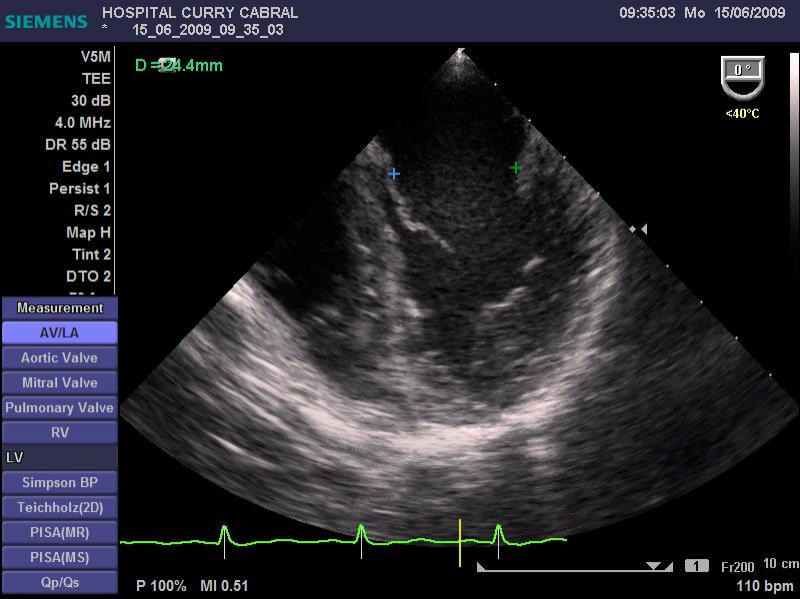
Measurement of mitral valve orifice.

**Fig. (3):**
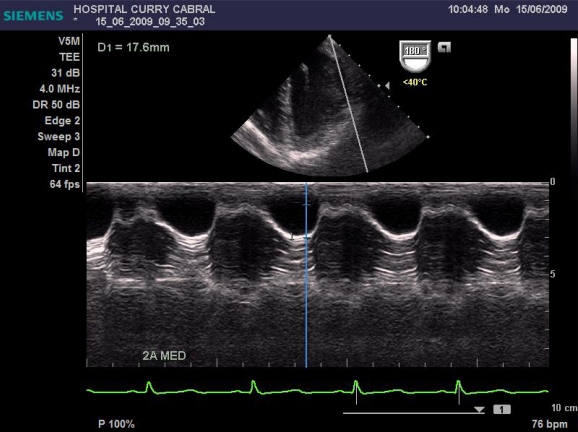
Measurement of the MAPSE.

**Fig. (4):**
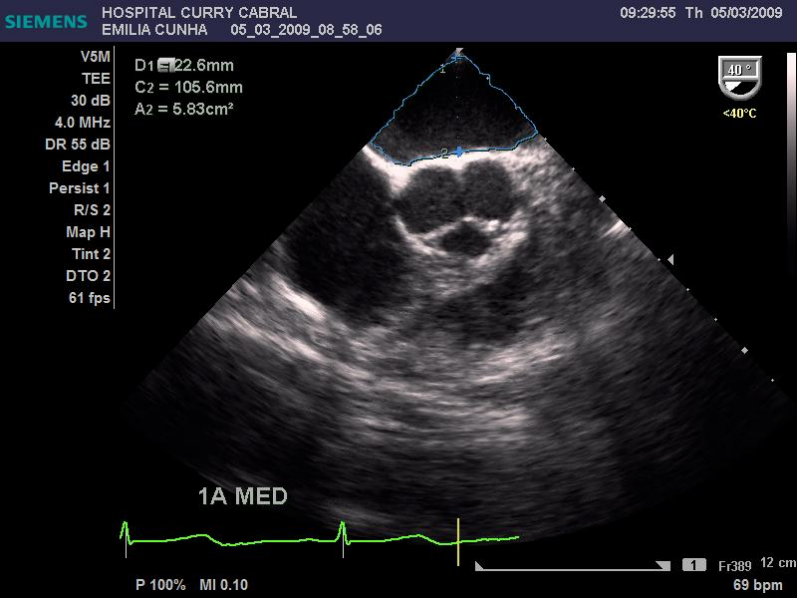
Measurement of the left atrial visible area and dimension.

**Fig. (5):**
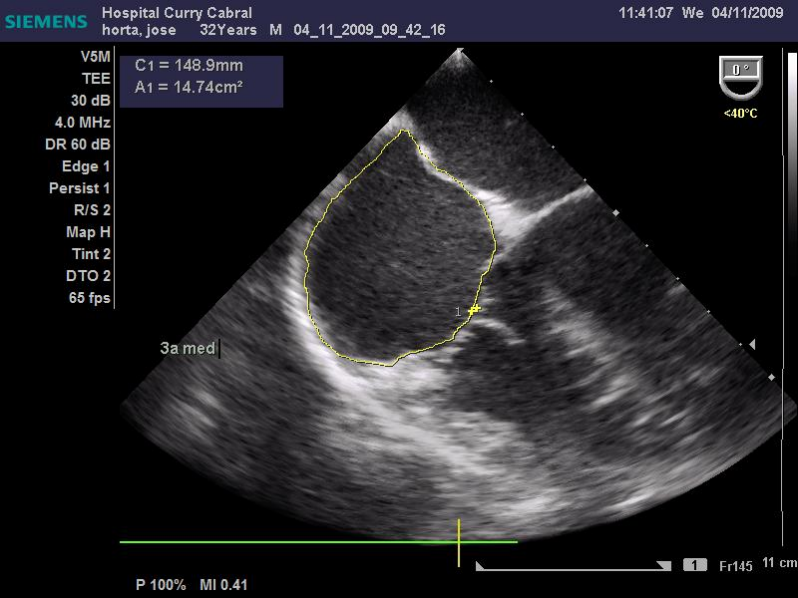
Measurement of the right atrial area.

**Fig. (6):**
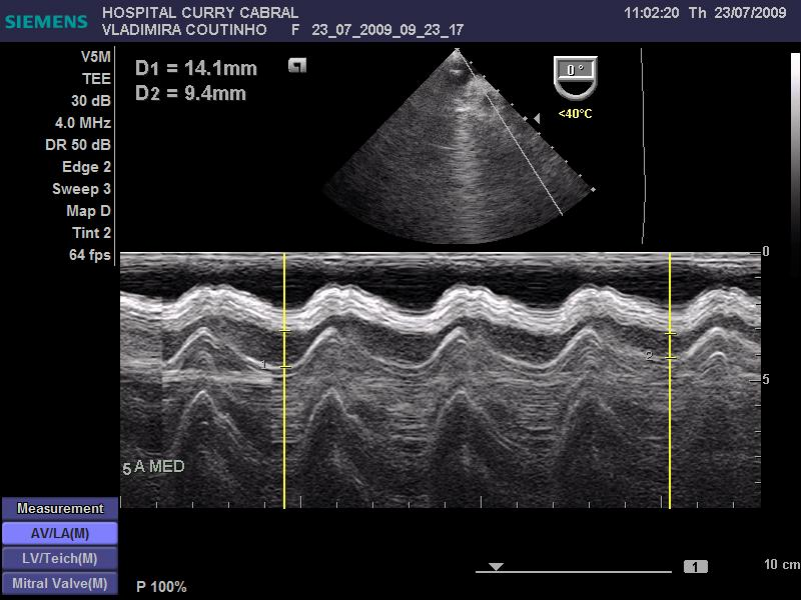
Measurement of the superior vena cava.

**Fig. (7):**
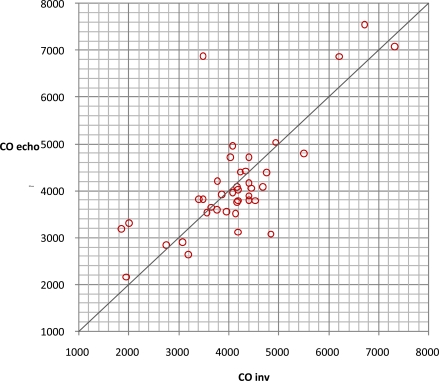
Correlation of cardiac output obtained by transesophageal echocardiography (CO echo) and by transcardiopulmonary thermodilution (CO inv).

**Fig. (8):**
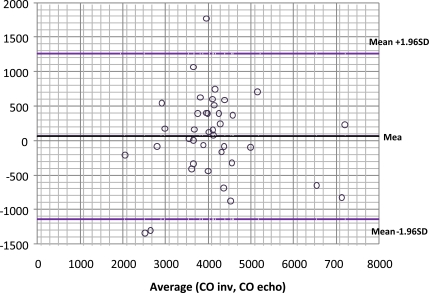
Bland-Altman plot comparing cardiac output obtained by transesophageal chocardiography (CO echo) and by transcardiopulmonary thermodilution (CO inv).
